# Supplementary material for: Detection of Rhodococcus fascians, the Causative Agent of Lily Fasciation in South Korea
Source: Pathogens. 2021 Feb 20;10(2):241. doi: 10.3390/pathogens10020241 (PMC7924060; doi:10.3390/pathogens10020241)
Supplement: Supplementary file 1 [file pathogens-10-00241-s001.pdf]

Supplementary Data

Detection of *Rhodococcus fascians*, the causative agent of lily fasciation in South Korea

Joon Moh Park et al.

Table S1. Sampling place of lilies located in Gangwon province of South Korea.

No	Location	Lily cultivar
1	X :128.172848, Y :37.3113234	Oriental hybrids
2	X :128.185448, Y :37.3120449	Asiatic hybrids
3	X :128.167835, Y :37.3066157	Oriental hybrids
4	X :128.267412, Y :37.2866898	Asiatic hybrids
5	X :128.229448, Y :37.5283488	Oriental hybrid ‘Siberia’

Table S2. List of oligonucleotide primers used in this study.

Primer name	Primer sequence (5'-3')	Target gene	Reference
27F	AGAGTTTGATCMTGGCTCAG	16S rRNA	Lane et al. 1991
1492R	GGTTACCTTGTTACGACTT		Turner et al. 1999
vicA44-F	TCCTATTCGATTTCGTCGAGAAG	*vicA*	This study
vicA737-R	GGGTCGATCTGGATCTCGAA		
fasD-F	ATTGTTGTTGCCGACCGTATC	*fasD*	This study
fasD-R	AAGGACGCCGTGCTCGACATAC		

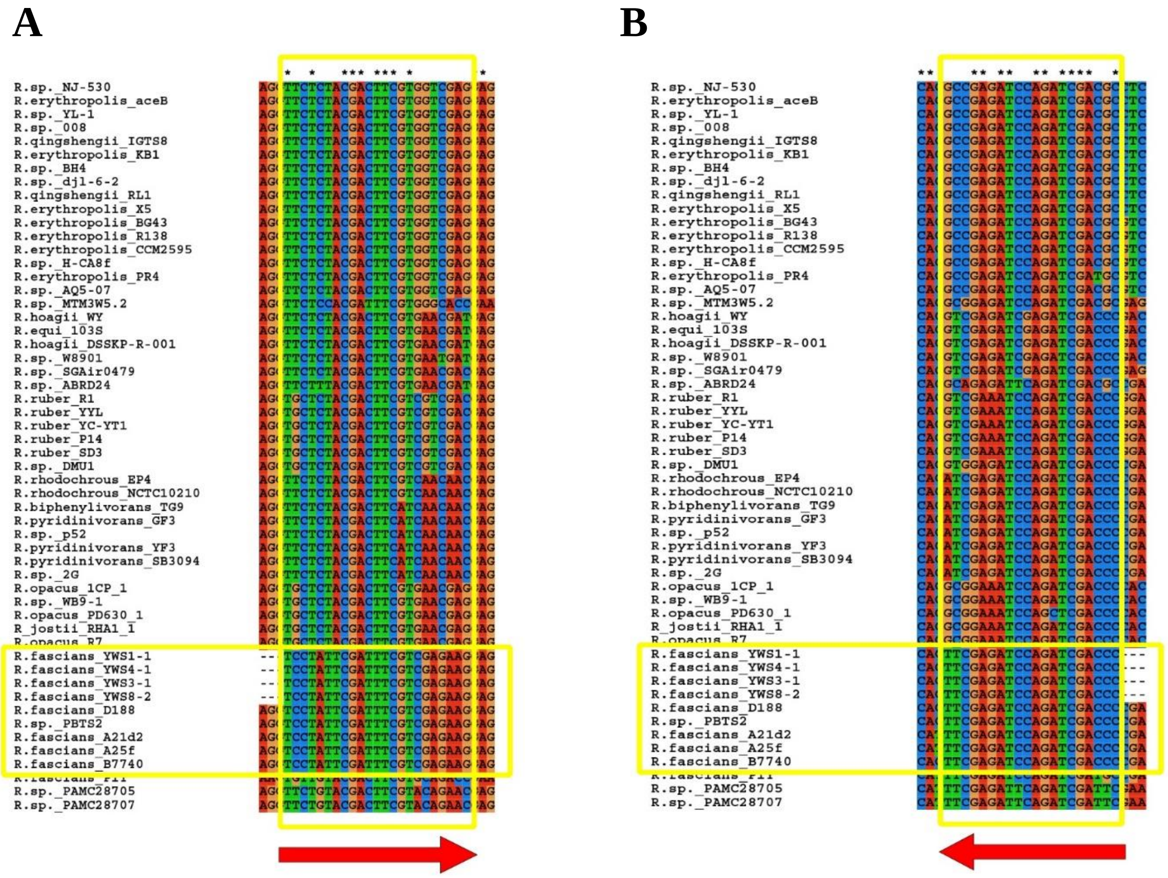

Figure S1. Multiple sequence alignment of the *vicA* genes of *Rhodococcus* species. The alignment was performed using ClustalW. The conserved region for *R. fascians* strains are represented by yellow rectangles. The red arrows indicate *vicA* primers developed in this study for diagnosis of *R. fascians.*

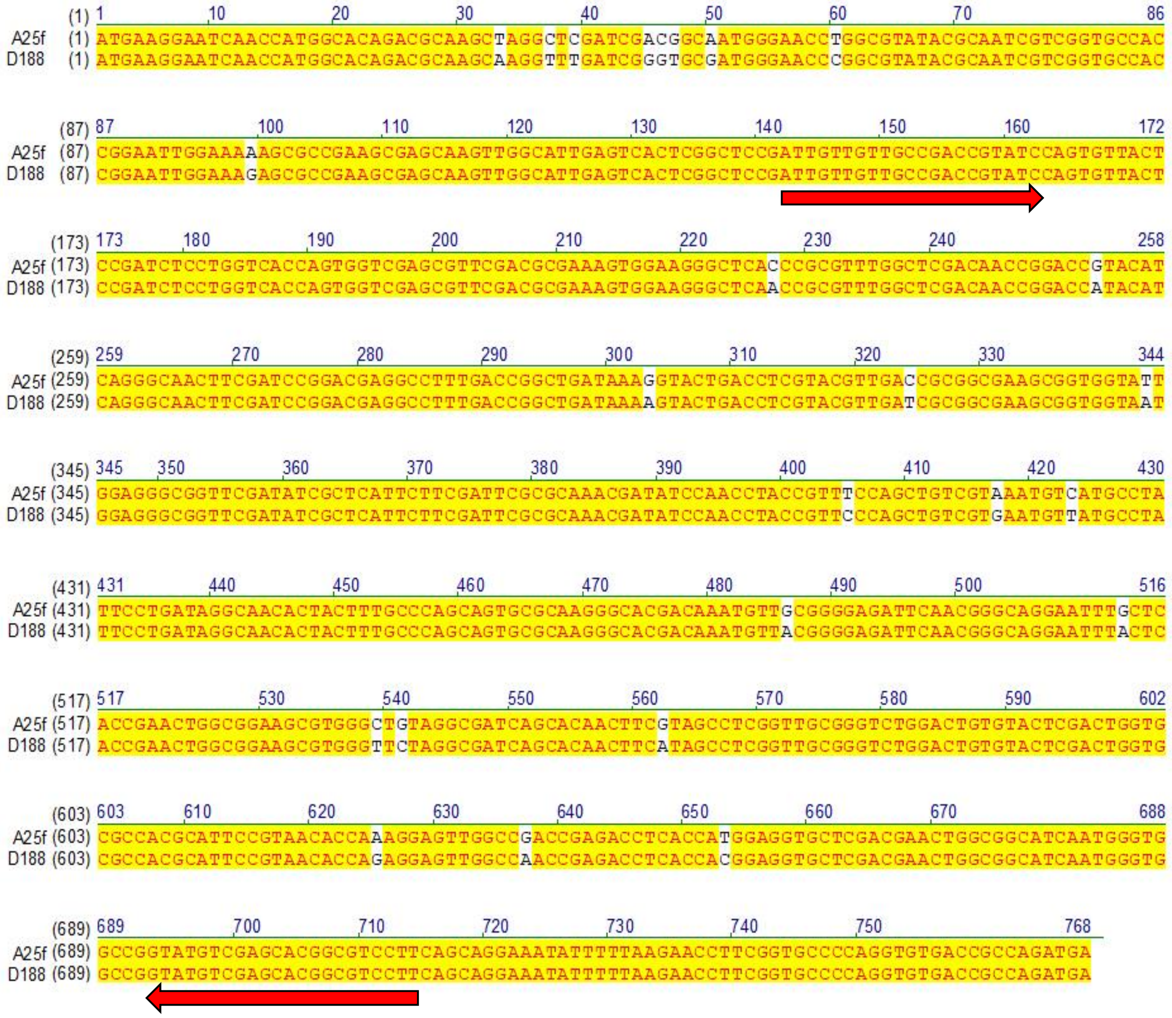

Figure S2. Design of PCR primers for *fasD* gene of *R. fascians*. Most *fasD* genes from pathogenic *R. fascians* can be classified into two types, A25f and D188. The pairwise alignment of two types of genes include from translational initiation codon to stop codon was performed to determine the conserved region. The red arrows indicate *fasD* primers developed in this study.

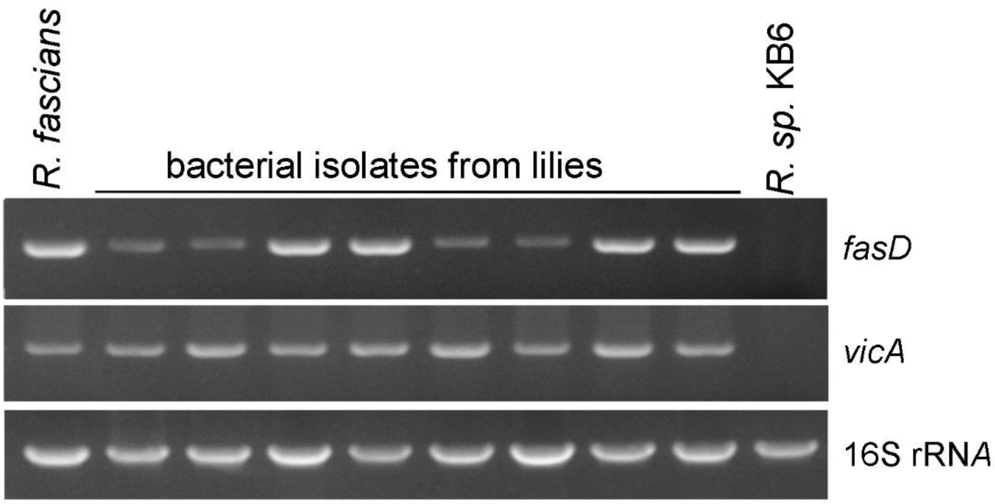

Figure S3. Representative PCR-based detection of *fasD*, *vicA*, and 16S rRNA genes of bacterial genomic DNA isolates. Total genomic DNA from bacterial isolates was used as template in PCR analysis. A previous isolate of *Rhodococcus* sp. (*R.* sp. KB6) was used as a negative control *for R. fascians*-specific primers for *fasD* and *vicA*. The 16S rRNA was used as an internal control. The amplicon size is 573 bp for *fasD*, 694 bp for *vicA*, and 1517 bp for 16S rRNA, respectively. *R. fascians* (Loewe Biochemica GmbH) was used for positive control of PCR.

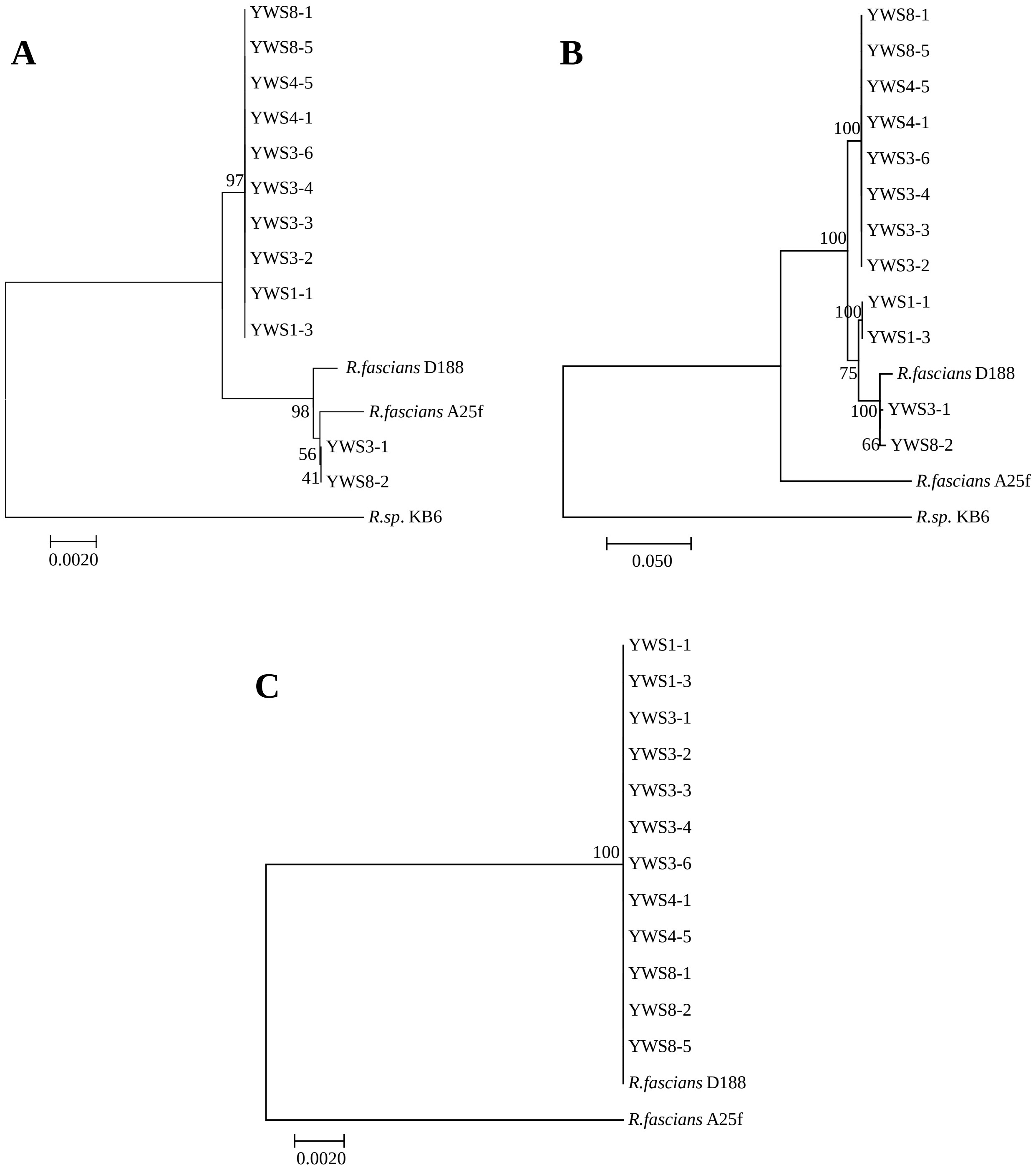

Figure S4. Phylogeney analysis of *R. fascians* YWS isolates from symptomatic lily plants. Phylogenetic trees were constructed based on nucleotide sequences of (A) 16S rRNA, (B) *vicA*, and (C) *fasD* using the Neighbor-Joining method by MEGA software. The reference sequences with Genbank accession numbers from *R. fascians* D188 and A25f, and *Rhodococcus* sp. KB6 were gathered from NCBI nr and wgs database. Accession numbers are followings: 16S rRNA gene for D188 (JMET01000045), A25f (CP049744), KB6 (LNAK01000053); *vicA* for D188 (CP015235), A25f (CP049744), KB6 (LNAK01000134); *fasD* for D188 (CP015236), A25f (CP049745).
